# Supplementary material for: The off-prescription use of modafinil: An online survey of perceived risks and benefits
Source: PLoS One. 2020 Feb 5;15(2):e0227818. doi: 10.1371/journal.pone.0227818 (PMC7001904; doi:10.1371/journal.pone.0227818)
Supplement: S1 File — (DOCX) [file pone.0227818.s001.docx]

**Advertisement**

**Recruiting – The off prescription use of modafinil and methylphenidate – all welcome!**

I am a PhD student at London South Bank University and I am researching the off prescription use of modafinil and methylphenidate, and also opinions on the use of cognitive enhancers. If you can spare 15-20 minutes, please complete this online survey (link found below) – you would be contributing to this investigation into use of these drugs, awareness of risks and harms and also opinions on the use of cognitive enhancers. You don’t need to have taken these drugs to participate, although anyone who has would also contribute greatly! This survey is completely confidential and anonymous so no details that could identify you will be recorded. As long as you are over 18 years of age and have an opinion about this topic you are most welcome to participate. This study is not restricted to any country and welcomes people from all countries to participate. Our findings will contribute to informing research on the extent of use of these drugs within the UK and internationally and will form the basis of a journal article that may be published in a peer reviewed journal.

The study has been approved by the Research Ethics Committee of London South Bank University (ref. 1626).

To participate, just follow this link: Smart Drug Survey
