# Supplementary material for: The off-prescription use of modafinil: An online survey of perceived risks and benefits
Source: PLoS One. 2020 Feb 5;15(2):e0227818. doi: 10.1371/journal.pone.0227818 (PMC7001904; doi:10.1371/journal.pone.0227818)
Supplement: S2 File — (DOCX) [file pone.0227818.s002.docx]

**List of subReddit forum sites**

**Reddit forum sites where the survey was advertised**

r/Nootropics

r/afinil

r/Stims

r/AskDrugNerds

r/StackAdvice

r/darknet

r/Drugs

r/UKUniversityStudents

r/UniUK

r/lifelonglearning

r/Scholar
