# Supplementary material for: The off-prescription use of modafinil: An online survey of perceived risks and benefits
Source: PLoS One. 2020 Feb 5;15(2):e0227818. doi: 10.1371/journal.pone.0227818 (PMC7001904; doi:10.1371/journal.pone.0227818)
Supplement: S3 File — (DOCX) [file pone.0227818.s003.docx]

**Demographics**

1. How old are you? (en*ter number*)
2. Are you male female (*tick boxes*)
3. What is your nationality *(free text box)*
4. What country do you live in? *(free text box)*
5. What is your highest level of education *dropdown menu: No formal qualifications,*

*Educated to age 16 (e.g. G.C.S.E.),*

*Educated to age 18 (e.g. A-level, High School, I.B.),*

*University Degree (BSc, BA),*

*Post-Graduate Degree (MSc, MA, PhD)*

1. Are you currently studying for a qualification? Yes No If yes, are you a university student? Yes No
2. Are you working at the moment? Yes, full-time paid Yes, part-time paid Yes, full-time voluntary Yes, part-time voluntary No If in employment, please state your job title or role *enter text*

**Drug awareness**

1. Are you aware of cognitive enhancing drugs? Yes (*yes leads to Medical health and drug use)*  No (*no leads to next question)*
2. If you could take a drug to increase your attention/concentration/memory, would you? Yes No Maybe

**Medical health and drug use**

1. Have you ever been diagnosed with a psychiatric condition? Yes (*leads to next question*) No (*leads to Q.14*)
2. What was the diagnosis?  *enter text (leads to next question)*
3. When was the diagnosis made? *Type year*
4. Are you still receiving treatment? Yes No
5. **Drug use** **Ever Used Used in the age when**

**last year first used**

Cannabis Yes No Yes No (type age)

Cocaine Yes No Yes No (type age)

Amphetamines (speed) Yes No Yes No (type age)

MDMA (ecstasy) Yes No Yes No (type age)

1. How often have you taken the drugs listed below in the last month? Select one answer for each drug taken.

**Every day/ once/twice once/twice less than once**

**Most days** **per week per month per month**

Cannabis

Cocaine

Amphetamines (speed)

MDMA (ecstasy)

1. Have you ever been treated for a drug or alcohol-related problem? Yes No

If yes, are you still receiving treatment? Yes No

**Modafinil use**

1. Are you aware of the cognitive enhancing drug, Modafinil (Provigil, Modalert)? Yes No (*no leads to methylphenidate use)*
2. Have you ever taken it? Yes No (*no leads to methylphenidate use)*
3. How many times have you taken modafinil? *dropdown scale: 1, 2 etc. to 10, then 10-20, more than 20*
4. How often do you take modafinil? *every day 3 or more times per week once or twice per week two or three times per month 6 times or less per year*
5. How much do you take at any one time?

L*ess than 100mg 100mg 200mg more than 200mg 400mg more than 400mg don’t know*

1. Do you usually use any other drugs at the same time? No Yes If so name them and specify level of dose (*free text box)*
2. How did you hear about modafinil?

*News article chat room friend online pharmacy dealer academic paper/lecture medical professional other please specify (free text box)*

1. How do you get modafinil (tick all applicable)

*GP prescription someone else’s prescription friend dealer online pharmacy*

*other please specify (free text box)*

1. Why do you take it? *To work longer hours for attention and focus to get more done for exams night work to think more clearly other please specify (free text box)*

**Risk awareness**

1. What immediate positive effects do you experience from modafinil? (tick all applicable)

*Increased energy ability to focus clarity of mind motivation confidence alertness increased productivity increased concentration improved reasoning increased creativity enhanced mood more outgoing/extraverted improved ability to enter a ‘flow-like’ state appetite suppression none*

*other please specify (free text box)*

1. What longer lasting (i.e. once the drug has worn off) positive effects do you experience from modafinil? (tick all applicable)

*Increased energy ability to focus clarity of mind motivation confidence alertness increased productivity increased concentration improved reasoning increased creativity enhanced mood more outgoing/extraverted improved ability to enter a ‘flow-like’ state appetite suppression none*

*other please specify (free text box)*

1. What immediate negative effects do you experience from modafinil? (tick all applicable)

*Anxiety insomnia diarrhoea dizziness nausea headache indigestion/acid reflux inflammation of the nose abnormal heart rhythm low blood pressure cannot empty bladder chest pains chills confusion depression fast heart beat mood changes problems with vision throat irritation dry mouth tremor vomiting loss of appetite other please specify (free text box) none*

1. What longer lasting negative (i.e. once the drug has worn off) effects do you experience from modafinil? (tick all applicable)

*Anxiety insomnia diarrhoea dizziness nausea headache indigestion/acid reflux inflammation of the nose abnormal heart rhythm low blood pressure cannot empty bladder chest pains chills confusion depression fast heart beat mood changes problems with vision throat irritation dry mouth tremor vomiting loss of appetite other please specify (free text box) none*

1. Do you feel dependent on modafinil? Yes No
2. How much modafinil do you think it is safe to take at any one time? None *50mg 100mg 200mg 400mg more than 400mg*
3. Do you think you would you be putting yourself in danger if you took modafinil

*every day 3 or more times per week once per week twice per month 6 times or less per year*

no danger

**Methylphenidate use**

1. Are you aware of Methylphenidate (Ritalin, Rubifen, Concerta)? Yes No (*no leads to PRMQ Questions*)
2. Have you ever taken it? Yes No *(No leads to PRMQ Questions. Yes for either leads next question*)
3. How many times have you taken methylphenidate? *dropdown scale: 1, 2 etc. to 10, then 10-20, more than 20*
4. How often do you take methylphenidate? *every day 3 or more times per week once or twice per week two or three times per month 6 times or less per year*
5. How much do you take at any one time? *10mg or less 20mg 30mg 40mg 50mg 60mg more than 60mg don’t know*
6. How much do you take in any full day? *10mg or less 20mg 30mg 40mg 50mg 60mg more than 60mg don’t know*
7. Do you usually use any other drugs at the same time? No Yes if so name them and specify level of dose (*free text box)*
8. How did you hear about methylphenidate?

*News article chat room friend online pharmacy dealer academic paper/lecture medical professional other please specify (free text box)*

1. How do you get methylphenidate (tick all applicable)

*GP prescription someone else’s prescription friend dealer online pharmacy*

*other please specify (free text box)*

1. Why do you take it? *To work longer hours for attention and focus to get more done for exams night work to think more clearly other please specify (free text box)*

**Risk awareness**

1. What immediate positive effects do you experience from methylphenidate?

*Increased energy ability to focus clarity of mind motivation confidence alertness increased productivity increased concentration improved reasoning increased creativity enhanced mood more outgoing/extraverted improved ability to enter a ‘flow-like’ state appetite suppression none*

*other please specify (free text box)*

1. What longer lasting (i.e. once the drug has worn off) positive effects do you experience from methylphenidate?

*Increased energy ability to focus clarity of mind motivation confidence alertness increased productivity increased concentration improved reasoning increased creativity enhanced mood more outgoing/extraverted improved ability to enter a ‘flow-like’ state appetite suppression none*

*other please specify (free text box)*

1. What immediate negative effects do you experience from methylphenidate?

*Anxiety insomnia diarrhoea dizziness nausea headache indigestion/acid reflux inflammation of the nose abnormal heart rhythm low blood pressure cannot empty bladder chest pains sweating confusion depression fast heart beat mood changes aggressive behaviour throat irritation dry mouth tremor vomiting loss of appetite other please specify (free text box) none*

1. What longer lasting (i.e. once the drug has worn off) negative effects do you experience from methylphenidate?

*Anxiety insomnia diarrhoea dizziness nausea headache indigestion/acid reflux inflammation of the nose abnormal heart rhythm low blood pressure cannot empty bladder chest pains sweating confusion depression fast heart beat mood changes aggressive behaviour throat irritation dry mouth tremor vomiting loss of appetite other please specify (free text box) none*

1. Do you feel dependent on methylphenidate?  *Yes No*
2. How much methylphenidate do you think it is safe to take at any one time? none *5mg 10mg 20mg 30mg 40mg 50mg 60mg more than 60mg*
3. Do you think you would you be putting yourself in danger if you took methylphenidate

*every day 3 or more times per week once per week twice per month 6 times or less per year*  *no danger*

**The Prospective and Retrospective Memory Questionnaire (PRMQ) items**

The following questions are about minor memory mistakes which everyone makes from time to time, but some of them happen more often than others. We would like you to tell us how often these things happen to *you*. Please indicate this by ticking the appropriate box. Please make sure you answer all of the questions even if they don’t seem entirely applicable to your situation.

1. Do you decide to do something in a few minutes’ time and then forget to do it? Very often Quite often Sometimes Rarely Never

1. Do you fail to recognise a place you have visited before? Very often Quite often Sometimes Rarely Never
2. Do you fail to do something you were supposed to do a few minutes later even though it is there in front of you, like take a pill or turn off the kettle? Very often Quite often Sometimes Rarely Never
3. Do you forget something that you were told a few minutes before? Very often Quite often Sometimes Rarely Never
4. Do you forget appointments if you are not prompted by someone else or by a reminder such as a calendar or diary? Very often Quite often Sometimes Rarely Never
5. Do you fail to recognize a character in a radio or television show from scene to scene? Very often Quite often Sometimes Rarely Never
6. Do you forget to buy something you planned to buy, like a birthday card, even when you see the shop? Very often Quite often Sometimes Rarely Never
7. Do you fail to recall things that have happened to you in the last few days? Very often Quite often Sometimes Rarely Never
8. Do you repeat the same story to the same person on different occasions? Very often Quite often Sometimes Rarely Never
9. Do you intend to take something with you, before leaving a room or going out, but minutes later leave it behind, even though it is there in front of you? Very often Quite often Sometimes Rarely Never
10. Do you mislay something that you have just put down, like a magazine or glasses? Very often Quite often Sometimes Rarely Never
11. Do you fail to mention or give something to a visitor that you were asked to pass on? Very often Quite often Sometimes Rarely Never
12. Do you look at something without realising you have seen it moments before? Very often Quite often Sometimes Rarely Never
13. If you tried to contact a friend or relative who was out would you forget to try again later? Very often Quite often Sometimes Rarely Never
14. Do you forget what you have watched on television the previous day? Very often Quite often Sometimes Rarely Never
15. Do you forget to tell someone something you had meant to mention a few minutes ago? Very often Quite often Sometimes Rarely Never

**OPINION QUESTIONS**

**Questions concerning the use of modafinil for cognitive enhancement**

**Please tick the statements that you most agree with**

*I feel it is entirely safe for people to take modafinil for cognitive enhancement strongly disagree disagree neutral agree strongly agree*

*I feel that taking modafinil for cognitive enhancement is dangerous strongly disagree disagree neutral agree strongly agree*

*If my peers were taking modafinil to help them achieve more I would feel pressured to do the same strongly disagree disagree neutral agree strongly agree*

*If my peers were taking modafinil to help them achieve it would not affect my choices strongly disagree disagree neutral agree strongly agree*

*People should be able to take modafinil to help them succeed if they want to strongly disagree disagree neutral agree strongly agree*

*Taking modafinil to get ahead gives those individuals an unfair advantage strongly disagree disagree neutral agree strongly agree*

*People who take modafinil for cognitive enhancement are cheating strongly disagree disagree neutral agree strongly agree*

*There is nothing wrong with people taking modafinil to get ahead strongly disagree disagree neutral agree strongly agree*

**Questions concerning the use of methylphenidate for cognitive enhancement**

**Please tick the statements that you most agree with**

*I feel it is entirely safe for people to take methylphenidate for cognitive enhancement strongly disagree disagree neutral agree strongly agree*

*I feel that taking methylphenidate for cognitive enhancement is dangerous strongly disagree disagree neutral agree strongly agree*

*If my peers were taking methylphenidate to help them achieve more I would feel pressured to do the same strongly disagree disagree neutral agree strongly agree*

*If my peers were taking methylphenidate to help them achieve it would not affect my choices strongly disagree disagree neutral agree strongly agree*

*People should be able to take methylphenidate to help them succeed if they want to strongly disagree disagree neutral agree strongly agree*

*Taking methylphenidate to get ahead gives those individuals an unfair advantage strongly disagree disagree neutral agree strongly agree*

*People who take methylphenidate for cognitive enhancement are cheating strongly disagree disagree neutral agree strongly agree*

*There is nothing wrong with people taking methylphenidate to get ahead strongly disagree disagree neutral agree strongly agree*

**Debrief**
