# Supplementary material for: The off-prescription use of modafinil: An online survey of perceived risks and benefits
Source: PLoS One. 2020 Feb 5;15(2):e0227818. doi: 10.1371/journal.pone.0227818 (PMC7001904; doi:10.1371/journal.pone.0227818)
Supplement: S1 Table — (DOCX) [file pone.0227818.s005.docx]

**S1 Table. Responses to “Please name them and specify the dose.”**

***Following the question: “Do you usually use any other drugs at the same time” (as modafinil)***

| 200mg modafinil As much caffeine as possible As much Marijuanna as possible |
| --- |
| 2400 mg piracetam, 100g l-theanine, 50-100g caffeine |
| 3 x Ibuprofen Codeine 200/12mg (600/36). Not for any psychoactive benefits, but for post-workout stiffness and pain. |
| Adderall 20-40 mg Ritalin 18-36 mg |
| alcohol marijuana |
| Alpha-GPC (Choline) - 300mg Omega 3 Fish Oil - 2g Phenylpiracetam - 100-200mg (Taken every other day when modafinil is taken) So one day on just modafinil, then a break, then one day on both modafinil and phenylpiracetam. |
| Aniracetam - 600mg Bromantane - 50 mg Piracetam - 600mg |
| Aniracetam, Alpha-GPC, Tianeptine, Semax, Selank |
| Bupropion XL 300mg po qam |
| Caffeine |
| Caffeine 100mg |
| Caffeine 100mg L-theanine 200mg |
| Caffeine 200 mg Sometimes Phenibut 1.5 grams |
| Caffeine 200mg, Cannabis, smoked |
| Caffeine every time, between 50-500mg, occasionally use about .6mg alprazolam towards the end of day to sleep/unwind, 25-50mg diphenhydramine if I need to sleep on modafinil because it's a first gen antihistamine with decent oral B/A and easily crosses the BBB so it tends to kill the high levels of hypothalimic histamine which blunts modafinil's effects. Phenibut and modafinil is one of my favorite combos, generally 1.5G phenibut with 2-300mg modafinil which keeps the social interactions fluid. |
| Caffeine, 200-400 mg |
| Caffeine, 200mg, L-Theanine, 400mg |
| Caffeine, phenyl-piracetam (and the hydrazide variant), aniracetam, and NSI-189. Have taken RAD-140, Ostarine, Clomid and many supplements with no negative interaction. |
| Caffeine, standard amount from store bought instant coffee or common energy drink e.g redbull |
| Caffeine, Theanine, some racetam 200, 400, depends on racetam |
| Caffiene 100mg |
| Caffiene 200mg L-Theanine 400mg St. John's Wort 3x200mg (spread out) |
| Cannabis - A few joints |
| Cannabis 1g |
| Cannabis 1g per day |
| Cannabis and alcohol |
| cannabis before i wanted to go to sleep after taking modafinil. couldn't sleep otherwise |
| cannabis-.5 grams |
| Cannabis, generally .5-.75 gs per session |
| Clonazepam, 2mg once a day. Caffeine, 200mg once a day. |
| Cocaine in assorted quantities, DXM 150mg, Heroin 250mg, Valium 10mg, Xanax 5mg. |
| Effexor 450mg |
| Enbrel 50mg weekly Cholesterol and blood pressure Blood thinner |
| Ethylphenidate 40mg insufflated |
| Excedrin - acetaminophen asprin and caffeine combo |
| F-phenibut 50mg, selegiline 5mg |
| Green tea L-theanine (~200 mg) |
| L theanine, Bacopa, Curcumin |
| Lamotrigine - daily, 25 mg Propranolol - PRN, 10-40 mg. Pregabalin - PRN, 50-200 mg. |
| LSD - 200ug Weed - ~3.5g |
| Marijuana at the end of the day |
| Metformin 2000mg |
| Nicotine - 20ml of 0.6% e-liquid Caffeine - 0.5g |
| Nicotine 4mg lozenges Dexamphetamine 5mg Caffeine 100mg |
| Nootropics - noopept, acetyl choline boosting supplements, racetams, caffeine, nootropic vitamins, intranasal insulin |
| Other Nootropics and supplements. Noopept 15mg, coluracetam 30mg, ginkgo recommended dose, ginseng 400mg. |
| oxiracetam x 400mg, semax x 200 mcg, alpha gpc x 300 mg, methylphenidate x 20 mg, acetyl-l-carnitine x 400 mg |
| phenylpiracetam, 250mg caffiene, 100mg l-theanine, 200mg |
| piracetam - 2g, oxiracetam - 1g, aniracetam - 1g, caffeine 3x a day ~30-50mg, perine tablet to increase modafinil effectiveness, coluracetam 3-6mg, Centrophenoxine |
| Piracetam 1.2g (three 400mg doses during the day) |
| Piracetam 400mg |
| Piracetam 800mg Caffeine 80-200mg |
| Piracetam, 4.8 g daily; Sulbutiamine, up to 1000 mg per day as needed |
| Pramiracetam, 200mg Alpha-GPC, 600mg Sarcosine, 2g (spread out through day) |
| Propranolol 40mg |
| T3 100mg Clenbuterol 50mg Testosterone 10mg Piracetam 800mg |
| weed few j |
| Xanex 25mg (I don't always mix these 2) |
