# Supplementary material for: The off-prescription use of modafinil: An online survey of perceived risks and benefits
Source: PLoS One. 2020 Feb 5;15(2):e0227818. doi: 10.1371/journal.pone.0227818 (PMC7001904; doi:10.1371/journal.pone.0227818)
Supplement: S2 Table — (DOCX) [file pone.0227818.s006.docx]

**S2 Table. FULL LIST OF POSITIVE AND NEGATIVE EFFECTS INCLUDING FREE TEXT ANSWERS**

| **Positive effects** | |
| --- | --- |
| Increased energy | improved reasoning |
| ability to focus | increased creativity |
| clarity of mind | enhanced mood |
| motivation | more outgoing/extraverted |
| confidence | improved ability to enter a ‘flow-like’ state |
| alertness | appetite suppression |
| increased productivity | none |
| increased concentration |  |

| **Free text answers for immediate positive effects** |
| --- |
| calm, energetic but not stimulated, just relaxed and alert |
| Can't sleep |
| Desire for processes (like lectures) to go faster leads to multitasking and less actual absorption of knowledge |
| Primarily for the first several hours of modafinil's subjective effects my social interactions become very easy to get through. |
| To be honest when I first started taking the tablets second year of university, it seemed like a wonder drug - felt like I was so much more involved and interested in lectures - answering questions making more of an effort understanding things quicker, committing concepts to memory faster. Eventually though it seems to have worn off, perhaps it was a placebo effect not really sure to be honest. Now it just feels like I am able to stay awake for longer periods of time. |
| calm, energetic but not stimulated, just relaxed and alert |

| **Free text answers for longer-lasting positive effects** |
| --- |
| Better memory |
| Decreased ability to sleep and eat |
| Fall asleep when I hit the pillow |
| I feel detached sometimes. |
| I reap the rewards of the work I've completed. |
| Increased memory |
| wakefulness |

| **Negative effects** | |
| --- | --- |
| Anxiety | chills |
| insomnia | confusion |
| diarrhoea | depression |
| dizziness | fast heart beat |
| nausea | mood changes |
| headache | problems with vision |
| indigestion/acid reflux | throat irritation |
| inflammation of the nose | dry mouth |
| abnormal heart rhythm | tremor |
| low blood pressure | vomiting |
| cannot empty bladder | loss of appetite |
| chest pains | none |

| **Free text answers for immediate negative effects** |
| --- |
| After a tolerance reset I experience a headache or two, as well as itchiness and dry mouth and itchy eyes a bit from the histamine. This passes, and usually indicates consumption of too large an initial dose after 2-5 days off of the drug. |
| Bad breath |
| Brain fog |
| Dehydration |
| Fatigue and aches |
| I can always tell when I have hit the crest in the modafinil curve when the chest pressure increases and breathing becomes a conscious process rather. I wouldn't go as far as to classify it as breathing difficulty, but it affects breathing. |
| I feel slightly itchy on modafinil (pretty sure it increases histamine, maybe due to this?) But only if I have taken it for more than a day in a row |
| I get insomnia, but it's not negative, it's one of the main reasons I use modafinil. Loss of appetite definitely occurs, but I have a very low appetite normally, so that's not entirely because of modafinil. Vision issues are because I'm often focusing at my laptop for hours without sleep, so I get very dry eyes. |
| Inability to focus |
| Irritability, emotional 'edginess'/hypersensitivity. |
| It tastes terrible going down. No matter how hard I try to avoid contact with my mouth, I always get some. |
| Itching of the skin |
| Mild, non-irritating rash |
| mouth sores |
| Not often but when I lie down at night it can be difficult to catch my breath |
| Rash |
| The most immediate negative effect is usually within 30 minutes or so the need to urinate - it's seems to dehydrate and dry mouth, so I end up drinking more water and as a result have to go urinate. More of an inconvenience than a negative effect. |
| Too much sweating, back pain |
| Vomiting is only sometimes. Unsure what causes it. |
| When taking more than approximately 300mg I have experienced over stimulating effects similar to a large amount of Caffeine, even when taken without Caffeine. Symptoms such as being jittery and even reducing work capabilities by being too over stimulated to concentrate. |

| **Free text answers for longer-lasting negative effects** |
| --- |
| Can get very hungry, although I'm always hungry. |
| Difficulty focusing with daily use |
| Exhaustion |
| eyes fatigue dry eyes eye irritation |
| fatigue |
| getting to sleep if i take it late in the day |
| Headache (sometimes) |
| If taken more than once a week. I develop 2-3 small red spots on my arms. Hence I keep the frequency to maximum once a week (if needed) |
| long term memory negatively impacted |
| Low energy. The low energy and depression are symptoms I already had, I just forgot about having to deal with them. |
| Not insomnia, but sleep less |
| Rash |
| tiredness |
| Tiredness and sleepiness. The positive effects barely last an hour, even when taking more than twice the recommended dosage. |
| Usually I like to take modafinil in threes. Once in the morning, then again in the evening to keep me going through the night, and the final one early on the second morning to make sure I'm not too tired to work the second day. When the modafinil wears off I'm obviously incredibly tired, and will feel lethargic even after sleeping 12 hours, which isn't so fun. On the bright side I usually relax at the end of the second day with cannabis, which works really well with weak modafinil and sleep deprivation. I've had some very strange auditory and visual hallucinations as a result of that, which I quite enjoyed. |
| Worse lack of motivation that ever before - i.e. at present, all motivated incidents in daily life are a consequence of having had Modafinil. If I don't have Modafinil, then I am unable to get things done at all. |
