# Supplementary material for: The off-prescription use of modafinil: An online survey of perceived risks and benefits
Source: PLoS One. 2020 Feb 5;15(2):e0227818. doi: 10.1371/journal.pone.0227818 (PMC7001904; doi:10.1371/journal.pone.0227818)
Supplement: S3 Table — (DOCX) [file pone.0227818.s007.docx]

**S3 Table. Timeframe and Frequency of modafinil use post-hoc within-subjects t-test and Cohen’s d results**

| ***Frequency of modafinil use*** | ***Immediate & Longer-lasting effects*** | | | | | |
| --- | --- | --- | --- | --- | --- | --- |
|  | ***Immediate*** | ***Longer-lasting*** | ***t*** | ***df*** | ***p*** | ***d*** |
| **Every day** | 5.64 (2.14) | 3.15 (2.05) | 6.91 | 25 | < .001 | 1.36 |
| **Three or more days/ week** | 5.44 (1.96) | 1.92 (1.49) | 14.00 | 65 | < .001 | 1.73 |
| **Once or twice/week** | 5.22 (1.86) | 2.17 (1.31) | 11.76 | 51 | < .001 | 1.63 |
| **Two or three times/month** | 4.74 (2.09) | 1.61 (0.92) | 10.15 | 37 | < .001 | 1.65 |
| **Six times or less per year** | 3.93 (1.56) | 1.50 (0.89) | 8.39 | 36 | < .001 | 1.38 |
