# Supplementary material for: The off-prescription use of modafinil: An online survey of perceived risks and benefits
Source: PLoS One. 2020 Feb 5;15(2):e0227818. doi: 10.1371/journal.pone.0227818 (PMC7001904; doi:10.1371/journal.pone.0227818)
Supplement: S4 Table — (DOCX) [file pone.0227818.s008.docx]

**S4 Table. Log-transformed data for 2 x 5 x 5 ANOVAs**

| **Main effect / Interaction** | ***F*** | ***df*** | ***df error*** | ***MSE*** | ***p*** | η_p_^2^ |
| --- | --- | --- | --- | --- | --- | --- |
| Main effect of frequency of modafinil use on the number of effects reported. | 5.53 | 4 | 214 | .53 | <.001 | .094 |
| Main effect of timeframe on modafinil use | 465.28 | 1 | 214 | .281 | <.001 | .685 |
| Main effect of perceived effects of modafinil | 375.49 | 1 | 214 | .414 | <.001 | .637 |
| Interaction between timeframe and frequency of modafinil use | 3.92 | 4 | 214 | .281 | .004 | .068 |
| Interaction between perceived effects and frequency of modafinil use | 2.491 | 4 | 214 | .414 | .044 | .044 |
| Interaction between timeframe and perceived effects of modafinil | 174.99 | 1 | 214 | .197 | <.001 | .450 |

**Respondents (N = 219)**
