# Supplementary material for: The off-prescription use of modafinil: An online survey of perceived risks and benefits
Source: PLoS One. 2020 Feb 5;15(2):e0227818. doi: 10.1371/journal.pone.0227818 (PMC7001904; doi:10.1371/journal.pone.0227818)
Supplement: S5 Table — (DOCX) [file pone.0227818.s009.docx]

**S5 Table. Perceived benefits and risks and frequency of modafinil use post-hoc within-subjects t-test and Cohen’s d results**

| ***Frequency of modafinil use*** | ***Perceived benefits and risks*** | | | | | |
| --- | --- | --- | --- | --- | --- | --- |
|  | ***Benefits*** | ***Risks*** | ***t*** | ***df*** | ***p*** | ***d*** |
| **Every day** | 6.77 (3.29) | 2.00 (1.43) | 7.13 | 25 | < .001 | 1.40 |
| **Three or more days/ week** | 5.53 (2.51) | 1.83 (0.91) | 12.00 | 65 | < .001 | 1.48 |
| **Once or twice/week** | 5.47 (2.36) | 1.92 (1.10) | 9.90 | 51 | < .001 | 1.37 |
| **Two or three times/month** | 4.74 (2.28) | 1.61 (0.71) | 8.98 | 37 | < .001 | 1.46 |
| **Six times or less per year** | 3.8 (1.71) | 1.64 (0.74) | 6.92 | 36 | < .001 | 1.13 |
