# Supplementary material for: The off-prescription use of modafinil: An online survey of perceived risks and benefits
Source: PLoS One. 2020 Feb 5;15(2):e0227818. doi: 10.1371/journal.pone.0227818 (PMC7001904; doi:10.1371/journal.pone.0227818)
Supplement: Attachment — Submitted filename: Response to Reviewers.docx [file pone.0227818.s010.docx]

Rachel Teodorini,

Division of Psychology,

School of Applied Sciences,

London South Bank University,

103 Borough Road,

London,

SE1 0AA, UK.

Tel : +44 (0)20 7815 5431.

Fax : +44 (0)20 7815 8099.

Email [teodorr2@lsbu.ac.uk](mailto:teodorr2@lsbu.ac.uk)

15^th^ October 2019

Dear Dr Hashimoto

**Re. Manuscript ID PONE-D-19-19392 entitled “The off-prescription use of modafinil: An online survey of perceived risks and benefits”**

Thank you for your email on the 5^th^ September detailing the reviewers’ comments on the submission. We were excited to hear that our research has aroused the interest and engagement of your two experts and that you are willing to consider a revised resubmission.

We have read through all of the feedback and found it to be very constructive. We have made what we believe to be the necessary modifications and improvements to the original manuscript in line with the comments provided by the reviewers.

As requested, we have provided a marked up copy of the manuscript highlighting changes made to the original version and an unmarked version of the manuscript.

**Addressing comments/suggestions from reviewer 1:**

Reviewer #1: General Comments
The authors have presented interesting descriptive data regarding the use of modafinil as a cognitive enhancing medication. The background information given in the introduction was well written and clear.
The aims and hypotheses seem unclear throughout, mostly in the results section, where a series of results is given without much explanation in the introduction or methods about the purpose of these analyses.

***We thank the reviewer for raising this issue. The aims have now been more clearly presented in the introduction and have addressed the results for demographics, motivations for using modafinil, perceived harms and access to modafinil.***

Apart from the descriptive statistics, the statistical analysis in this paper is unclear. A thorough review of the statistics is required by the authors, making sure that appropriate statistical tests are used for different types of data.

***We appreciate the reviewer’s point. We have now reviewed our statistical tests thoroughly and we have added an explanation for our treatment of the number of effects as continuous rather than categorical and an explanation of how Cohen’s d was calculated. We hope that our explanations meet with the reviewer’s approval.***

Specific Comments
Introduction
- Some of the references for modafinil are outdated

***Reference 5 (now 9) and 17 (now 26) have been replaced.***

– and reference 6 seems incorrect

***We thanks the reviewer for spotting this error. Reference 6 (now 20) has now been corrected.***

- Does the study consider use of armodafinil or just modafinil? Armodafinil is the r-enantiomer of racemic modafinil and often the two are used interchangeably or quite similarly. Can the authors comment on this?

***Although armodafinil may also be used as a cognitive enhancer it does have a different profile and may be experienced differently to modafinil. It may also be less well known. Therefore to include armodafinil would provide less specific data. To ensure that respondents understood which drug the questionnaire was asking about, brand names were also provided (Provigil, Modalert). This is now stated in the Methods section under the Modafinil subheading.***

- The start of the final paragraph on page 7 is repetitive

***The start of this paragraph has been reworded to avoid repetition.***

- Were the aims and hypotheses made a-priori or post hoc? Was there a primary hypothesis?

***This study was exploratory in nature given the lack of literature available specifically on perceived effects of modafinil outside of laboratory conditions. The aims were a priori and the specific hypotheses were data-driven.***

Methods
- The design section describes the statistical methods – I believe this section should report the study design, not stats used.

***The details of statistics used have now been moved to the Analysis subsection of the Method section.***

- Why did the authors exclude those who were prescribed modafinil? They could have been prescribed it and still be using it for cognitive enhancement.

***We appreciate the reviewer’s point. We excluded those who were prescribed modafinil as our focus was purely on the off-prescription use, perceptions and understandings of the risks and harms associated with this unsupervised use in addition to understanding the profile of this kind of modafinil user. Those who have a prescription are, to some extent, under medical supervision and are prescribed the legitimate medication whereas those who purchase via online pharmacies, the dark web or from another person may not be receiving modafinil and may not be aware of the strength, purity, drug interactions etc.***

Additionally, why did people complete the survey if they were not modafinil users – could they have been past users? I would like to see some information about these people who were excluded as it may be of interest

***We thank the reviewer for asking this question. This survey was the first study of my PhD and therefore sought to obtain data from a wide variety of CED users. However, this paper has focused on modafinil in order to provide information specifically on the use of this drug, which appears to be the most commonly used CED.***

- The Analysis section requires further description of the statistical tests performed

***We thank the reviewer for this advice. We have now added an explanation for our treatment of the number of effects as continuous rather than categorical and an explanation of how Cohen’s d was calculated. We hope that the reviewer feels this is adequate.***

- Under “Procedure” the specific ethics committee details and approval number should be given

***The ethics committee details and approval number have now been included in the “Procedure” section.***

Results
- In Table 1 the authors have presented both benefits and risks together. I think it would be of more interest to separate the effects into the two as it may be different and of interest to readers to present it that way?

***We appreciate the reviewer’s comment. Table 1 was presented to show the main effect of ‘frequency of use’. It should therefore be collapsed across any other factors included in the ANOVA. The difference between benefits and risks is illustrated in Fig 2 and we hoped that this would be sufficient for readers to see.***

Can the authors justify their hypothesis here? The hypothesis described in the introduction does not match the presentation of the results here.

***We thank the reviewer for highlighting this. The data presented in descriptive form only have been clarified in the new paragraphs on the aims of the study. Each of the inferential tests relates to the hypotheses as follows. Hypotheses 1 – 3 were tested by the 2 x 2 x 5 ANOVA, hypothesis 4 was tested by the Mann-Whitney tests. A parallel structure was used in both the statement of hypotheses and the results section.***

- The statistics used to describe the effects of frequency of use and number of effects are unclear. This is ultimately categorical data which seems to have been initially represented like it is continuous, then a series of comparisons have been made and p values given, but no comparative statistic given. The statistical methods should be better described in the methods and should be clearly reported.

***We thank the reviewer for identifying this issue. This has now been explained more clearly in the Analysis subsection of the Method section which states:***

***“***As the highest number of positive effects selected by any respondent was 14 (1) and the highest number of negative effects selected was 7 (1), not including options of ‘none’ and ‘other’, these data were treated as continuous. The large number of cells with uneven cell sizes would have prevented a coherent analysis of categorical data.**”**

***. In addition and for clarity two extra columns have been added to Table 1 to include maximum and minimum overall number of effects.***

At the end of page 15 there are a series of values given as plurals. Perhaps this information would be better placed in a table in the supplementary material if need be.

**This has now been placed in a table in the supplementary material.**

- It is unclear how the Cohen’s d values listed at the top of page 16 were calculated.

***We thank the reviewer for pointing this out. Cohen’s d calculations have now been explained in the Analysis section.***

Again this appears to be count data, and should not really be converted to mean values.

***We appreciate the reviewer’s point. However, as explained earlier, due to the high number of positive and negative effects respondents had ticked, treating the data as categorical would have resulted in a vast number of categories to analyse. It therefore seemed a better approach to treat the data as continuous.***

The Cohen’s d values reported reflect very large effect sizes, but this is not commented on further.

***We appreciate the reviewer’s point. The effect sizes are large as they are all generated from highly significant differences identified in the post-hoc t-tests. In situations like this, a large effect size should be expected. Cohen’s d has now been presented alongside the t-tests in supplementary tables 2 and 3 in order to clarify how this relates to interpretation of the 2x5 ANOVA. A comment regarding this has also been added to paragraph 4 of the discussion.***

- Figures should be used to support the presentation of data to support the main findings. Neither of the figures match hypotheses outlined in the introduction.

***We appreciate the reviewer’s point. However, we believed that Fig 1, the interaction between immediate and longer-lasting effects and frequency of use of modafinil, links to the first hypothesis (more frequent use of modafinil would be associated with greater perceived benefits). This interaction illustrates that the more frequently modafinil is used, the greater the number of effects and as the respondents reported more benefits (mean = 4.99, SE = 0.14) than risks (mean = 2.07, SE = 0.10) this matches the first hypothesis. This can also be seen in Fig 2, the interaction between numbers of benefits and risks and frequency of use of modafinil. This interaction shows that both immediate and longer-lasting benefits were higher than immediate and longer-lasting risks. We feel that Fig 2 therefore also matches the first hypothesis.***

Discussion
- The initial paragraph of the discussion should describe the main results and then start to place them in context of what is known. The discussion of limitations should be saved until later in the discussion section.

***We thank the reviewer for making this point. Limitations have now been moved to later in the discussion section, the main results are now described in the first paragraph and then placed in context.***

- Again the primary and secondary hypotheses should be outlined clearly here with reference to the results.

***We appreciate the reviewer’s opinion, but having reviewed the discussion section thoroughly, we feel that the hypotheses have been clearly outlined with reference to the results.***

- The authors acknowledge that selection of a sample from groups of people who discuss the use of the drug as a cognitive enhancer online may be different to normal users. Do we know how many people use the drug as a cognitive enhancer?

***As with all illicit drug use, the proportion of the population who use modafinil off-prescription is unknown. Obtaining a representative sample of illicit drug users is always difficult and data obtained will be biased according to the sampling methods used. In this case, the bias is towards users of drug use forums*.**

**Addressing comments/suggestions from reviewer 2, Dr Hironaka:**

Reviewer #2: This is an interesting study on the off-prescription use of modafinil. The data presented here would provide valuable information for considering the pros and cons of modafinil as a cognitive enhancing drug (CED).

The paper is well written in general, but a few modifications would help readers to understand the significance of the data more straightforwardly. Please consider the following points:

1) Introduction
This section is a little bit too long and the specific topics of modafinil and CEDs in general are mixed together.
For example, after the introduction on modafinil, concomitant use of CEDs (general) and illicit drugs appeared (p. 4, paragraph 2, ‘Strong associations…’). Then the story goes on to the effects of modafinil in laboratory settings. After that, problems about studies on CEDs in general appear again (p. 5, the last paragraph, ‘However, these studies…’ and subsequent paragraphs, p. 6. ‘Survey data…’, ‘Although the perceiver effects of CEDs’).

***We thank the reviewer for highlighting this. The introduction has now been changed to start with CEDs and then move on to modafinil. We have also reduced this section down and hope that the reviewer feels that it is now a good length.***

I would like authors to emphasize more straightforwardly (1) why you focused on modafinil,

***We have now emphasized why we focused on modafinil. This can be found towards the end of the first paragraph. The sentence begins“Although the perceived effects of CEDs in everyday settings*…”.**

and (2) why you employed an online survey.

***We have now emphasized why we employed an online survey and this can be found towards the end of the paragraph starting “However, these studies demonstrate the effects…”***

2) Results

2-1) Could you show several typical examples of perceived risks and benefits? This would help readers to determine what kind of benefits and risks the users perceived.

***We have now added more examples of perceived risks and benefits and we hope the reviewer feels that this is adequate.***

2-2) A total of 15 positive effects and 24 negative effects were listed (p. 11) but the reported numbers of overall effects (both positive and negative) ranged from 2.27 to 4.39 (Table 1). Does this mean the majority of users did not perceive any effects? Am I right? If so, please mention this briefly.

***We thank the reviewer for asking this question. The majority of respondents reported at least one effect. Out of 219 respondents only 2 reported no effects.***

Plus, the rationale for combining both positive and negative effects should be explained.

***We thank the reviewer for making this point. This has now been explained in the Analysis sub-section of the Method section. In addition and for clarity two extra columns have been added to include maximum and minimum overall number of effects.***

3) This section is also a little bit too long. To organize this section better, please consider the following points:

3-1) Considerations on the biochemical mechanisms of action of modafinil and their relevance to motivation for use (p. 21, paragraphs 3 and 4, ‘Deficiencies in DA, NE…’, ‘As reported by Cools et al…’) are over-speculation and should be more concise.

***We thank the reviewer for making the point of over-speculation. We have now added in parethasis “while highly speculative…” in paragraph 6. We have also removed the paragraph discussing Cools et al.’ work.***

Plus, please consider referring to action on the histaminergic system. This seems important for night-time use of modafinil.

***We appreciate the reviewer’s point. This has now been added to the introduction in the second paragraph.***

3-2) As the authors mentioned, this study has several limitations, such as gender bias, source of information from drug forums, and lack of questions on attentional problems. In this manuscript, these are scattered in several paragraphs. I think it is better to create a new paragraph and compile these topics. Plus, in my opinion, several important questions were missing, such as status of cigarette smoking, use of other CEDs, and perceived levels of academic achievement. Could you include these limitations?

***We thank the reviewer for making this point. The limitations have now been brought together in the final paragraphs before the Conclusions section and nicotine and use of other CEDs have also been mentioned. However, questions were asked on levels of academic achievement and these data were presented in the ‘demographics’ subsection of the results section.***
